# Cerebral protection in aortic arch surgery: systematic review and meta-analysis

**DOI:** 10.1093/icvts/ivac270

**Published:** 2022-11-17

**Authors:** Vivek Patel, Vicente Orozco-Sevilla, Joseph S Coselli

**Affiliations:** Division of Cardiothoracic Surgery, Michael E. DeBakey Department of Surgery, Baylor College of Medicine, Houston, TX, USA; Department of Cardiovascular Surgery, Texas Heart Institute, Houston, TX, USA; Division of Cardiothoracic Surgery, Michael E. DeBakey Department of Surgery, Baylor College of Medicine, Houston, TX, USA; Department of Cardiovascular Surgery, Texas Heart Institute, Houston, TX, USA; Department of Cardiovascular Surgery, CHI St. Luke’s—Baylor St. Luke’s Medical Center, Houston, TX, USA; Division of Cardiothoracic Surgery, Michael E. DeBakey Department of Surgery, Baylor College of Medicine, Houston, TX, USA; Department of Cardiovascular Surgery, Texas Heart Institute, Houston, TX, USA; Department of Cardiovascular Surgery, CHI St. Luke’s—Baylor St. Luke’s Medical Center, Houston, TX, USA

**Keywords:** Aortic arch aneurysm, Aortic dissections, Thoracic aorta

Cerebral protection during aortic arch surgery is based on reduction in metabolic demand (hypothermia) and delivery of metabolic nutrients (antegrade or retrograde cerebral perfusion techniques) to reduce the risk of stroke [1]. The risk of neurologic deficit remains 5–10%, despite the advent of adjunctive cerebral perfusion techniques in the 1990s [1, 2]. In a noble effort to determine the most effective type of adjunctive cerebral perfusion, Abjigitova *et al.* conducted the largest systematic review on this topic, a meta-analysis of 222 studies involving 43 720 patients [3].

The authors conclude that unilateral antegrade cerebral perfusion (ACP) had a lower mortality (6.6%) and stroke rate (4.8%), whereas bilateral ACP (9.1% mortality, 7.3% stroke), retrograde (7.8%, 6.4%) and deep hypothermic circulatory arrest without adjunctive perfusion (9.2%, 6.3%) had higher rates of mortality and stroke. However, these conclusions must be tempered with the following considerations. The data are diverse because it is mostly from observational studies, which include multiple procedures (hemiarch, total arch), indications for surgery (dissection, aneurysms) and experience (smaller and larger centres across the world). Importantly, there is no consensus on the criteria for selecting antegrade, bilateral antegrade, retrograde or deep hypothermic circulatory arrest without cerebral perfusion. Confounding considerations (pre-existing cerebrovascular anatomy, history of stroke, anticipated complexity of the procedure) may have led to the selection of 1 cerebral perfusion technique over another. The lowest temperature and total time of cerebral perfusion for each technique were also incomplete across the studies. Unfortunately, without this level of granularity, it becomes quite difficult to conclusively determine if 1 technique is indeed superior to another. Accordingly, the authors are careful to not directly compare 1 technique to another.

Nonetheless, the meta-analysis adds to the literature by correlating the findings of similar studies by Angeloni *et al.*, Lou *et al.* and our group [4–6]. We applaud the authors for reviewing 222 studies with 43,720 patients to gain insights into the trends regarding this important topic and providing real world data for the currently used techniques of cerebral perfusion. Any type of cerebral perfusion (antegrade or retrograde) is preferable compared to having no cerebral perfusion. There is a trend towards less use of retrograde cerebral perfusion [3]. Unilateral ACP is a relatively simple, reproducible technique which has gained popularity. However, since 6–17% of the adult population has an incomplete circle of Willis, our preferred technique is bilateral ACP, especially when a circulatory arrest time of greater than 30 min is anticipated due to the complexity of the procedure, i.e. total arch replacement [6]. Notably, Angeloni *et al.* [4] and Preventza *et al.* [6] and found no statistically significant difference in the mortality or stroke rate for unilateral or bilateral ACP. A direct comparison of the cerebral perfusion techniques would require analysis of the same type of procedure (aneurysm or dissection), evaluation of the patient’s cerebrovascular anatomy, evaluation of the total cerebral perfusion time and postoperative evaluation with neuroimaging in addition to the presence of transient or permanent neurologic deficits. Given the complexity and emergent nature of the procedures that require cerebral perfusion, such a study would be quite challenging to execute.

## Data Availability

No new data were generated or analyzed in support of this research.
